# Principes généraux de Physiologie-Pathologique, coordonnés d'après la Doctrine de M. Broussais

**Published:** 1821-04

**Authors:** 


### CRITICAL ANALYSES

OF

### RECENT PUBLICATIONS, IN THE DIFFERENT BRANCHES OF MEDICINE AND SURGERY,

### In the Literature of Foreign Nations.

---

Παῖδες ἄρα
Ανδράσιν, ὃ πατραῖς ἀνδρες, ἀγαλλόμεθα.

---

*Principes généraux de Physiologie-Pathologique, coordonnés d'après la Doctrine de M. Broussais.* Par L. J. Bégin. 8vo. pp. 390. Mequignon-Marvis, à Paris, 1821.

IN the preceding article on the subject of this work, we took into consideration some of the principles most obviously connected with the development of morbid actions in the human body: we shall now pass in review, in a similar manner, several of the most important phenomena of disease, and endeavour to indicate how far they may be illustrated by means of the principles above alluded to, without entering more deeply into the history of their origin than is conformable with the researches of our author, and the ordinary habits of reflection of medical practitioners.

Mr. Bégin commences his disquisition on maladies in general, with the proposition that all diseases have their origin from more or less severe "lesion" of one or other of the "organs" of the body: by which statement, he means to indicate that there are no original diseases of the system generally, and that many of the symptoms in those appearing to be so extensively seated, are only effects or accidental contingencies of the essential organic lesion. This proposition will by many men be considered so obvious an inference from the best physiological, as well as etiological, views, as not to require an author now to produce any arguments in its support; and Mr. Bégin would probably have done no more than simply state it, had there not been recent instances of attempts to render prevalent some vague, or more properly, absurd, notions of certain diseases (idiopathic fever, as they say,) having their original seat in no one part especially, but in the whole system generally; a proposition which precludes all reasoning on the nature of the diseases said to be of this species; for it is impossible to draw any inferences respecting their etiology, when we have no ideas on which any reasonings can be founded.

All the local lesions which produce the apparent external phenomena, by the aid of which we recognize those diseases, do not leave traces, after death, in the part they have affected. Morgagni, Mr. Bégin remarks, and several other physicians whose accuracy of observation and due qualifications cannot be doubted, have stated that they have seen instances of patients dying after having experienced the symptoms of apoplexy or of pleurisy, without the body, after death, presenting the slightest sign of lesion of the brain or of the pleura. But these cases do not lead to the inference that diseases may exist without derangement in the vitality and texture of the organs: they only show that these derangements are sometimes so inconsiderable as to be dissipated, or not to be apparent, after death. The redness of the cheeks and tongue, which is sometimes produced by gastro-enteritis; peripneumonia; erysipelas, and other cutaneous inflammations, though very severe during life, often disappear instantly after death; and why may we not conclude that the same results may take place in inflammation of the internal organs?

After the establishment of the proposition above stated, a question, Mr. Bégin says, the solution of which is fundamental in pathological physiology, is this: "In how many different ways may the living tissues be morbidly altered?" Notwithstanding the objections which have been raised against it, and the partial modifications which it has suffered, the fundamental proposition on which ***Brownism*** rests, Mr. Bégin says, has been religiously preserved; and Professor Broussais himself, who is considered as the most strenuous adversary of that doctrine, in some degree conforms to it. "The ***pathologic lesions**** of the diverse tissues, says the author of the new medical doctrine, are characterized by the diminution or the augmentation in their vital phenomena. For us, the first of these states is an ***ab-irritation***, the second a ***super-irritation,*** or ***morbid irritation.***" On this point Mr. Bégin very properly remarks, that a consideration which presents itself before the question can be discussed, is, that the tissues which Bichat regarded as simple are very complicated, and should be analyzed by the pathologist. There enters, for example, in the composition of the mucous membranes, capillary blood-vessels, arterious and venous; cerebral nerves, and nerves of the ganglionic system; mucous follicles; exhalant and absorbent vessels; and it is obvious that especial lesion of each of those elementary textures will produce particular and peculiar effects in the organ in which it is seated, as well as in the remote parts with which it is related. With respect, too, to the therapeutical indications founded on the same principles, there are certain agents which seem to modify with more predilection one of those elementary textures than another. Thus, some purgatives often confine their agency to the production of a more abundant secretion of mucous fluid; certain stimulants excite more expressly than others the nervous sensibility, whilst many substances of the same kind act with more energy on the capillary blood-vessels. These distinctions may seem to be minute, but they are well-founded, and present some important therapeutical indications. They elucidate phenomena which are constantly witnessed in practice, and, amongst others, the effects, in a manner elective, of a given stimulant which cures, whilst another stimulant, not apparently more energetic, would have certainly been deleterious. Thus, for example, there is a certain degree of gastric inflammation which is removed by a dose of tartar-emetic, and which

* We cite here the terms of Mr. Bégin.

would have been aggravated by wine or ether: the tartar-emetic, wine, and ether, are all stimulants; and the first, if applied to the skin in a healthy state, would excite more inflammation in it than either of the latter. A multitude of examples of a similar kind will readily present themselves to the reflective observer.

'Besides these considerations, Mr. Bégin adds, that we reasonably doubt if these notions of augmentation and diminution of action being the only modes of essential disease, "the simplicity of which is so seductive, and which the physician uses with so much assurance at the bed-side of the patient, are conformable with the course of nature;" and it is, he continues, "not without alarm that we can see all diseases reduced to two classes; the materia medica to two kinds of medicines; and general therapeutics to two curative indications."

"We cannot conceive, we must confess, what can be the nature of the vital actions which should not consist in depression or exaltation of the powers; but this motive is not sufficient for their rejection, if accurate observations attest their existence: this relation of more or less is not the only one which may exist, either between bodies, or their qualities, or the actions they execute. Does it not appear, for example, that syphilitic irritation, which has the same seat as scrofula, differs from the latter only in degree? The different species of herpes, although seated in the same order of vessels, present peculiar diversities which are not explicable by diversities in the violence of the irritation. Let us however observe, that, in proportion as physiological pathology is improved,—as we study with more attention the diseased tissues, and take into consideration the modifications which diversities of age, sex, and temperament, effect in the morbid phenomena,—we see diminish the number of diseases to be attributed to different species of augmentation or diminution of the vitality of the organs. The time will perhaps arrive at which the two principles of the Scotch physician will be demonstrated to be exact in all cases; but hitherto the evidence does not appear to be complete: new researches are necessary, in order to elucidate this point of doctrine."

We have cited thus copiously from the work before us, in order that we might enable the reader to form an idea of the scope of Mr. Bégin's reflections in this instance: the examination of the subject has been pursued to its utmost depth by only one author, whom we should incessantly quote, were we to do justice to his merits.

However it may be with regard to the subject of the foregoing discussion, Mr. Bégin says "it is indubitable that a very great majority of diseases consists in exaltation or diminution of vital action, and that one or other of these alterations always effects certain organs, which then produce in the economy very various sympathetic effects, which characterize, externally, the different diseases." The views here indicated are, perhaps, as penetrative as any that can be taken with our present knowledge of the human economy; and, what is more worthy of consideration, they are probably sufficiently so for the purposes of general use, where more profound information and nicer distinctions might not be often available.

"Are the diseases which consist in *irritation** more frequent than those of which debility is the first cause?" is a question next noticed by Mr. Bégin: it is one, he says, that merits all the attention of physicians, for it must lead to very different practical results, according to the manner in which it is solved. Broussais, though he has designated many of the diseases which he considers to consist in *irritation*, has not yet expressed his opinions on this point in a comprehensive manner: Mr. Bégin endeavours to supply the deficiency here alluded to.

"It would seem, on a first consideration," Mr. Bégin says, "that vital action is as susceptible of diminution as of exaltation. It would be possible even to sustain this proposition in a general manner; and many physicians would not only have faith in it, but would demonstrate that the former of these deviations is of more easy and frequent occurrence than the latter. However, when we study facts, when we observe a great number of patients, and we arrive thus to the source of our knowledge, we soon acquire the most complete conviction that irritations constitute alone almost the entire demesne of pathology. Look over the nosologic tables; study the phenomena of diseases; open dead bodies: every where, in books, at the bed-side of patients, and in the amphitheatres, you will find proofs of the correctness of this assertion." These three (or properly two) sources do not present such demonstrative proof of Mr. Bégin's assertion as he conceives; or they only show that certain phenomena, which we confound altogether under the name of irritations, most generally accompany the progress of diseases. The appearances found on the examination of dead bodies, are often only very remote effects of the first morbid change in the constitution of the parts affected, to which we must advert if we are to have correct notions of the nature of the disease; and with respect to the symptoms, during life, of *irritations*, it yet remains to be proved that they are indications of an exaltation of vital action. But let us admit, as a matter of convenience, that irritation is an exaltation of vital action, or is essentially allied with such an exaltation, and that the development of irritation is the first or most important morbid change in the diseases which are accompanied with, or ordinarily characterized by, it:—let us admit thus much, and see how Mr. Bégin supports his proposition, that such diseases constitute alone almost the entire demesne of pathology. "What has led systematic physicians to erroneous conclusions," Mr. Begin says, "is their appreciating the force of the irritated organs by that which is possessed by the external parts; or, rather, they most commonly are ignorant of what organs are affected, and, seeing the limbs enfeebled, they infer that the disease is owing to debility, which has struck, as they say, the whole organization."—"These heresies," he

* The word *irritation*, employed in a general manner, according to Mr. Bégin, "signifies an augmentation of action of the capillary vessels or of the nerves which enter into the composition of parts. But, whenever it is necessary to give more precision to language, and to designate the texture especially affected, it becomes convenient to employ the following expressions: *sanguine irritation*, *lymphatic irritation*, *nervous irritation*, &c."

adds, are no longer admitted by enlightened practitioners; and their discredit is such, that criticism should disdain to combat them." This emphatic, rather than forcible, reasoning, leads him to assert, in continuation, that "observations, well directed, demonstrate *then* that the most part of affections consist in irritation of the organs; and my task would be confined to the announcement of this general result of the best-directed clinical investigations, and to cite MORGAGNI, BICHAT, PROST, DUPUYTREN, LAENNEC, and BROUSSAIS, who have rendered it incontestible, if it were not indispensible to theory to trace the series of physiological phenomena that explain the fact above stated." This is effected by Mr. Bégin in the following manner; omitting here only some details not of essential importance to the disquisition.

The nutritive actions of composition and decomposition preside over the existence of animals and vegetables. In order that a body having life may execute all the actions necessary for this function, it is requisite that it be excited; if it ceases to be thus affected, it languishes and perishes. Two classes of agents, indispensible for the maintenance of life, and which may become powerful causes of disease, are the excitants of the organic movements; one of which consists of matters that stimulate the economy, without presenting to it any thing which is capable of increasing the quantity of its liquid or solid materials;* the other of substances which provoke the exercise of its functions, at the same time that they furnish it with the matters which repair the losses it suffers. A subdivision of the agents which excite the animal economy, is that into such as stimulate the whole system, and such as exert their influence only on certain parts of it. The general stimulants are light, caloric, electricity, oxygen, aliments of good quality, a due abundance of blood properly endowed with nutritive materials. The energetic, long-continued, and united, action of those different excitants, augments the force and vivacity of the vital actions, and thus disposes all the organs to excessive excitement; and the state of the economy which results from this violent stimulation (which is most commonly experienced in the age of adolescence, and most remarkbly in the nations which inhabit the hotter regions of the globe,) is sometimes carried to such a degree that the subject of it, tormented by a superabundance of force, instinctively gives himself up to the most fatiguing exercises, and to the most destructive excesses, in order to dissipate this exuberance of vitality, which torments him beyond endurance.

The superabundance of very stimulating nutritive materials may arrive at such a state as will determine, in the most sensible parts, and especially in the mucous membranes and in the blood-vessels, an irritation which is followed by disturbance of the circulation and the other functions, and which constitutes that variety of inflammatory fever that Sauvages called plethoric.

Different effects are produced when the non-nutritive stimulants are

---

* This reasoning is shown to be incorrect in a work lately reviewed in this Journal. See vol. xliv. pp. 40—42.

the only species which act with an excess of power. The organs then are incessantly stimulated, without the losses produced by the vital movements being properly repaired; the nervous susceptibility then becomes extremely developed; the body becomes emaciated and enfeebled to the utmost degree. Such of the people of Asia and Africa as indulge in a continual abuse of opium, coffee, and in undue development of certain passions, as venus nimia, &c., and who feed on aliments having but little nutritive qualities, present examples of the effects of the inordinate use of non-reparatory excitants on the animal economy.

The privation of the two classes of general stimulants above mentioned, leads to a diminution of the vital powers in all the textures. The nutritive elaborations languish and become less complete; the organs, imperfectly nourished, are incapable of fulfilling their functions in the ordinary manner; the colourless fluids predominate over the red blood, and the latter itself is of a less intense colour, and contains less of nutritive matter than in the more healthy state. The results of the want of these general stimulants may be observed in cold, humid, and marshy, countries, where the inhabitants are nourished with a meagre and insalubrious diet. Persons in this condition are equally deprived of moral and physical energy,* and their vital actions preserve in their diseases a remarkable degree of sluggishness and debility, whilst the sympathetic movements in them are deprived of energy and vivacity. All the parts of the body are not, however, equally debilitated in these subjects. "The weakness resides almost exclusively in the exterior parts and in the least important organs: in proportion as the forces diminish, life becomes concentrated, in a manner, and retires towards the parts the most indispensable to the preservation of the individual; the principal focuses of the vital power absorb the last remains of the nutritive materials, and are the seat of the latest movements."

The superabundance or the penury of the general stimulants determine, in the whole economy, a relative excess of force or of more or less considerable debility. The too energetic action of those agents excites especially the nervous system; their privation debilitates the same parts, favours the elaboration of colourless fluids, and alters the composition of the blood. In the first case, there is an imminent disposition to irritation; but, all the organs possessing the degree of energy which is appropriate for them, and being habituated to a regularity and facility of action, there is established in the economy an "equilibrium," which the most immoderate abuse of excitants can hardly destroy. In the second case, the disposition to irritation is far from being abolished; it is perhaps more considerable† than in the

---

* We may refer the reader to the writings of Montesquieu, Kaims, Cabanis, and De Stael, for extensive disquisitions on this subject, and a due appreciation of the general statement of Mr. Bégin.

† This doctrine, of parts in a state of relative debility being *therefore* specially disposed to irritation, at the same time that irritation is regarded as an exaltation of vital action, is sadly in want of something like plausible arguments for its support: it is, however, a piece of doctrine that is now very popular.

other condition; the nervous mobility* favours "local concentrations," which take place with particular facility in the abdominal viscera: the "equilibrium" being but ill-established, or even broken in favour of those viscera, their lesion is the effect of the action of the most slightly energetic causes; though, if a person escape the influence of such causes, he may live in a languid state, and be even ready to perish, by the gradual extinction of his strength, without presenting any sign of local irritation, or any other "lesion" existing than his debility.

The most general, most active, and most efficacious, cause of excitation of the organs, is the exercise of the functions of those organs; an exercise which is provoked by the application of the stimulants which are proper to them: and, on the contrary, a want of exercise of them is the circumstance which most contributes to enfeeble them. Exercise determines in the vessels of the organs a more considerable afflux of fluids; their tissues, when excited by this cause, become more solid, more voluminous, and acquire an increased ability for action; but such effects take place only when the elements of nutrition are carried to the organs in a due abundance, and when they are of a good quality: without this last condition, the parts would only become enfeebled by their exertion.

It is to the alternative action of our organs that are to be attributed, almost exclusively, this "equilibrium" of the vital movements, and those regular and periodic returns of repose and excitement, that are observed during life. Those alternatives of action, their periodic returns, and the simultaneous excitement of two or more organs, rapidly become habitual when they have once been established, and constitute associations of functions and synergies, which play an important part in the development of the phenomena of diseases. Disease of one organ thus rapidly excites disease of other and remote organs, by an influence that is impalpable to the senses, but which we know, from its effects, to exist.

The inaction of one of our organs, by depriving it of the concentration of vitality which is the immediate effect of irritation, and by keeping absent from it the materials of nutrition, which are elsewhere directed, debilitates this organ, and insulates it, in a manner, from the rest of the economy; "but without any sympathetic effect in the other organs resulting from this inaction."† In order to recognize the correctness of this proposition, it is, however, necessary to distinguish, in diseases of debility, that which appertains to the local debility, from that which is produced by *the interruption of the functions of the enfeebled organ.* "All the sympathetic lesions are produced by the nervous system,"‡ and "it is not possible for them to be effected

* It should be remembered that Mr. Bégin has just said that the nervous system is especially enfeebled in this condition.

† This last proposition is very disputable, even with the modifications of it which immediately ensue. It is amply discussed in the Dissertation which gained the Jacksonian prize in 1813, and in several parts of the "General Indications which relate to the Laws of the Organic Life."

‡ This is a disputable proposition. If one branch of a polypus be rudely otherwise than by means of the influence of an organ which is itself primarily *irritated.*"* Thus, for example, the stomach may be in a state of debility, without any sympathetic effect in the system resulting from it.† But, in this case, the organ will effect an imperfect elaboration of the aliments it receives, and this imperfection will give rise to more or less secondary phenomena. "An organ simply enfeebled, is not painful; it cannot, consequently, transmit any excitation to other parts. The physiologist should regard it merely as a parasitic instrument, which lives at the expence of the economy, the mass of which is useless, but which exerts on the rest of the body no other deleterious influence than that which results from the cessation of its functions."

Such are the principles on which Mr. Bégin founds his conclusion that by far the greater part of diseases are constituted of *irritations*:‡ but there are, he says, other reasons from which it happens that, whatever may be the nature of diseases on their first development, physicians hardly ever have to treat any others than those of irritation. "Men are," Mr. Bégin says, "as it is well known, much more dis-

---

touched, another branch of it will, as an effect of this impression, be put into vital action: this effect cannot well be distinguished from those we term sympathies; and no nerves have been found in the polypus. A similar effect may also be witnessed in the sensitive plant. Some men, it is true—reasoning on the *petitio principii*, that no mode of sensibility can exist without nerves,—have inferred that even plants must possess nerves: but, if we are to contemn the evidence of our senses after this manner, we must resign all our pretensions to knowledge on any subject.

* This is a very interesting question in physiology: whether there be a communication of properties from one part of the economy—otherwise than from the blood and the nervous system, (which Mr. Bégin, from some inadvertency, we conjecture, neglects to notice, unless it may be considered that he has contemplated this in the effects of *the functions of organs:)*—to others, in the state of health, that is necessary to the preservation of this state; and, therefore, whether diseases may not arise in various parts, from an enfeebled organ not transmitting its ordinary influence, *as well as from a communication of* IRRITATION? We know, however, no positive arguments in favour of the affirmative; whilst the negative is supported by such as these,—that the rest of the economy will preserve its ordinary health after the loss of two or three limbs. Mr. Bégin's proposition must, therefore, be considered as highly probable.

† Mr. Bégin says, that the sense of weakness about the epigastrium, and the general languor which arises from it, (and which are removed by the smallest quantity of food or a single glass of wine,) from hunger, should not be considered as objectionable to his proposition: "it is incontestible," he says, "that there is then *pain* and irritation in the stomach, and that the general symptoms are produced by this pain, which is alleviated by food or drink:" but, if substances of too stimulant a kind, or such as are not proper for nutrition, are taken, this irritation is aggravated, and the general debility increased.

‡ This term is here used according to Mr. Bégin's application of it: that is, as designative of an exaltation of vital action. When we ourselves use this word, it should be understood that we employ it as a concise expression of our ignorance, when we suppose that some modification of vital action, of a morbid kind, exists, with the nature of which we do not pretend to be acquainted. We use the term excitement, to designate what we conceive to be a simple augmentation of vital action. We are not, however, satisfied that it is possible for such a mode of action to exist; yet it may be convenient to have a term for expressing an augmentation of action which is not accompanied with, or necessarily followed by, any sensible morbid effects.

posed to the abuse of stimulants than to endure their privation. *It is extremely rare that this privation is sufficiently complete to give rise to* [literally, to *determine*,] *diseases;** and when persons, even those the most exempt from prejudices, become sick, they at once resort to the use of the most active tonics or excitants. The labourer, oppressed by want, collects the last remains of his property, to procure himself wine, brandy, or other analogous liqours. Hot wine with sugar, punch, *vulneraries*, preparations of steel, elixirs of all sorts, sudorific infusions, compose the pharmacopœia of the more opulent persons. It is extremely rare, in the practice of medicine, either in hospitals or in the largest cities, to meet with diseases *free from all curative attempts*:† almost always have the subjects endeavoured to remedy them by the aid of stimulants. There results from this general practice, that, if the disorder has depended on a penury of excitants, it is rapidly dissipated, and the physician is not consulted. Professional aid is sought only when the affection has resisted the common *touchstone*, and when the patient has already aggravated the disorder by the incendiary agency of the most powerful tonics; and, solely because his advice is required, the practitioner should expect to have to treat a disease which consists in irritation of some one of the organs: examination but rarely controverts this presumption."

Mr. Bégin then sums up his inferences respecting the nature of diseases in general, in the following manner:

"1°. The excessive action or the privation of general stimulants, never provokes, in the economy, any thing more than states of force and of debility, respectively, which predispose to local diseases. 2°. Even in the case of a penury of stimulants, the weakness is greater in the exterior than in the central parts; and this circumstance favours the development of irritation of the latter. 3°. Local *ab-irritations* cannot excite any sympathetic phenomenon of re-action in remote parts. 4°. The absolute privation of food, and the use of substances not proper for nutrition, which are considered as the general causes of debility, provoke irritation of the digestive organs, which complicate and augment the state of debility of the other parts of the body. 5°. Although diseases originating from debility were more frequent than we can suppose them to be from the foregoing reasons, the physician would yet have but a very small number of them to treat, because patients almost always, before they resort to professional aid, adopt the use of stimulants, which cure them in cases of debility, but which, most commonly, aggravate irritation."

Mr. Bégin says that he should here have terminated what he had to say about diseases in general, if, after Brown, the existence of passive inflammations and hemorrhages had not been a point of doctrine "generally professed." The objectionable notion here indicated is by

* Mr. Bégin should read Dr. HARTY's History of the late Epidemy of Ireland.

† Mr. Bégin will excuse our altering his mode of expression in our translation. "Maladies *vierges* de toute tentative de traitement," is too metaphoric, too delicate for English sensibility, to permit us to adopt the expression, however highly we may ourselves admire and approve such an ornament of style in medical literature.

no means generally adopted amongst us, and those who profess it, after having considered what has been said on the subject by Dr. Parry and some other of our writers, would not, we conceive, have their faith destroyed by the arguments of Mr. Bégin. Professor Broussais' opinions on this subject, and his etiology of scurvy,—a disease which has been considered to present proof of the existence of passive hemorrhages,—have already been stated in this Journal. We shall, however, transcribe the paragraph which forms a conclusion to Mr. Bégin's discussion on these subjects: it presents in itself a summary of his opinions.

"Thus, on carrying our analysis to the utmost point, it is irritation that is the sole cause of the 'oscillation' and the 'equilibrium' of the vital actions; it is that which 'calls' the fluids, and which determines their afflux to this or that particular part; it is irritation which excites, or which exasperates, the motions of the capillary vessels, whether sanguineous, secretory, exhalant, or others, whatever may be the elaborations with which they are charged, and the composition of the fluids they prepare or contain. *Ubi stimulus, ibi affluxus*, and *Duobus laboribus simul obortis, vehementior obscurat alterum:* such are the bases of physiology and pathology. These axioms are, in medicine, of an importance equal to that of this proposition, that all bodies reciprocally attract each other, and that their attractive force is in a direct ratio of their masses, and in an inverse ratio of the square of their distances: a proposition which serves as the basis of all explanations in physics."*

* It would be very amusing, were not the objects of medicine of too serious a nature to allow of such a feeling being indulged when the best interests of that science are violated, to contemplate the diversities of opinion amongst men of eminent talents respecting even the very principles of pathology. Broussais and Mr. Bégin regard irritation as the foundation of almost, if not quite, all diseases; and irritation they consider to be an affection, originally, of the nervous system.* These are notions which have been designated by a modern English author, who was really a medical philosopher, as "either visionary or inapplicable, and which lead to practices tending equally to debase the moral character of mankind, to produce or perpetuate disease, and to discredit the medical profession."

* Mr. Begin says, in conformity with Broussais, that "the agents of irritations commence, in all cases, with agitating the nervous system, which seems to be especially destined to collect all impressions; it is only secondarily that the stimulation is communicated to the capillary vessels, and that the fluids are directed towards the part."

